# Nitrogen and phosphorus losses from paddy fields and the yield of rice with different water and nitrogen management practices

**DOI:** 10.1038/s41598-020-66757-5

**Published:** 2020-06-16

**Authors:** Dongliang Qi, Qixia Wu, Jianqiang Zhu

**Affiliations:** Engineering Research Center of Ecology and Agriculture Use of Wetland, Ministry of Education, Yangtze University, Jingzhou, Hubei 434025 China

**Keywords:** Wetlands ecology, Environmental impact

## Abstract

The assessment and control of losses of nitrogen (N) and phosphorus (P) from paddy fields is critical to improve the quality of water and atmosphere on earth. A field experiment was conducted to investigate the effect of three N managements (local common N fertilization practice, urea mixed with controlled-release N fertilizer, and optimized and reduced N fertilizer, designated CN, U + CRF and ON, respectively) on N and P losses through runoff and leaching from a paddy field, and yield of rice under shallow-irrigation and deep-sluice (SIDS) and continuous flooding irrigation (FI) in the Jianhan Plain of China in 2016. The results showed that, compared with FI, SIDS significantly reduced the frequency of irrigation and amount of irrigation water, resulting in an increase of 16.2% in rainfall use efficiency, and therefore, a reduction in the amount of surface runoff and water that had leached. This was responsible for the decreased total N (TN) and total P (TP) losses through runoff leaching under SIDS. The U + CRF and ON treatments resulted in a significant reduction in losses of TN through runoff and leaching and the loss of TP through leaching compared to CN. SIDS resulted in comparable or greater soil TN and TP contents in the 0–40 cm soil depths after rice harvest; N and P accumulation at the jointing, filling and maturity stages; and yield of grain compared to FI. Moreover, the U + CRF and ON improved or maintained accumulation of N and P and yield of rice compared to CN. Compared with FI coupled with CN, SIDS coupled with the U + CRF or ON treatments significantly reduced losses of N and P from paddy fields and enhanced or maintained the accumulation of N and P and yield of rice grains. In conclusion, SIDS coupled with the new N management could be an effective approach to reduce losses of N and P from paddy fields and would be a positive improvement for high yield of middle-season rice grains in the Jianhan Plain of central China and other regions with similar environments.

## Introduction

Rice (*Oryza sativa* L.) is one of the primary crops in the world and the foremost staple food in Asia, supplying 35–60% of the dietary calories consumed by more than three billion people^1^. In China, the average annual area of rice planted and total production had reached 30.1 million ha and 18.6 billion t in 2011, respectively^2^. Both the planting area and total production ranked first in the world^3^. However, the growth of rice requires a substantial amount of fresh water, so that the rice planting system accounted for 45–50% of total water consumption in China^4^. Moreover, a shortage of water resources is a serious problem in China, and its spatial and temporal distribution is extremely uneven^5^. The water scarcity is further strengthened by climate change, a limited supply of water, and the increasing water consumption by cities, industries and other sectors of the economy^6,7^. This has encouraged more researchers to develop novel irrigation strategies to improve crop water use efficiency (WUE), so that the sustainability of rice production could be assured^8,9^.

Various water-efficient irrigation management modes are currently practiced in different paddy fields in China, including alternate wetting and drying, shallow-irrigation and deep-sluice (SIDS), intermittent irrigation, controlled irrigation, flooding-midseason drainage-frequent water logging with intermittent irrigation, and semi-dry cultivation among others^10–13^. Among these irrigation methods, SIDS is considered to be an efficient irrigation method to maintain the yield of rice, while reducing no-point pollution and the amount of irrigation compared to continuous flood irrigation in the Jianghan Plain of China^13–15^. In SIDS, the precipitation is sluiced to some extent, and the field remains non-flooded unless cracks appear on the soil surface; thus, alternate wetting and drying cycles occur in paddy fields during the whole rice growing season^14^. Because of the high economic return and its ease of application, SIDS has been widely practiced in several provinces in China, including Hubei, Hunan, Jiangsu, and Anhui.

Nitrogen (N) fertilizer is another important input for intensive rice production^16^. The average yield of rice per unit area in China is 6.18 × 10^3^ kg ha^−1^, which was 65% higher than that of the average yield in world; the amount of N fertilizer applied for rice production in China accounts for 37% of the N fertilizer used for rice in the world^17^. Thus, the recovery efficiency of fertilizer N is only approximately 30%, which is approximately 40–50% lower than the world average^18^. Over and/or improper fertilization is a serious issue in intensive agricultural production areas in China, contributing to soil degradation, lake eutrophication, groundwater pollution, and the emission of ammonia and greenhouse gases^19–22^. The loss of nutrients from agricultural fields is one of the main contributors to these types of pollution^20,21^, particularly in the central and southern regions of China^23^. Additionally, N fertilizer is becoming expensive because of increasing demand for fossil fuels worldwide. Therefore, exploring effective measures to reduce nutrient losses from farmland, while maintaining crop yield has become a priority for the sustainable development of agriculture, particularly for rice production.

To not only favor the profitability of farmers but also reduce the negative environmental impacts, several researchers have tested different N management practices to improve N use efficiency (NUE)^7,18,23^. Some N saving methods (site-specific N management, balanced N fertilization, integrated N management, the use of nitrification/urease inhibitors and slow/controlled-release fertilizers) are already showing promise^2,23,24^. Recently, the use of controlled release N fertilizer has been found to be an effective way to increase the NUE and reduce N losses from paddy fields^23^. Moreover, a reduction in the level of N fertilizer supplied and amount of irrigation is the most effective measure to control nitrate leaching in cropland farming^25,26^. In addition, optimized fertilization could increase the NUE of rice^27^ and reduce the N and phosphorous (P) losses from source^19^. However, the effects of N-saving methods vary depending on different environmental conditions.

The key growth period of rice usually coincides with the plum-rain (a weather phenomenon characterized by continuous overcast and rainy weather) season in the Jianghan Plain of China, leading to more frequent surface and underground drainage occurring from paddy fields, and an indirect drought often happens after the plum-rain^28^. Moreover, earlier research has illustrated that soil nutrients and water availability are closely linked and mutually influence one another^29^. Fertilization and water management are two important factors that influence the migration and use efficiency of N and P in paddy fields^30^. Thus, it is necessary to explore an individual appropriate N application mode under water-saving irrigation based on specific weather conditions in the rice planting areas, such as the Jianghan Plain of China. In addition, the manner in which controlled release N fertilizer and optimized N management influence the losses of N and P from paddy fields and yield of rice has yet to be addressed under SIDS.

This study was designed to quantify the losses of N and P through leaching and runoff from a paddy field, N and P accumulation in rice, and yield of rice with controlled release N fertilizer and optimized N management under SIDS. Moreover, the contributions of various forms of N and P compounds to the total N and total P losses through leaching and runoff were quantified. The results should provide effective measures to reduce the losses of N and P in paddy fields and improve rice yield in the Jianghan Plain of central China and other regions with similar environments.

## Materials and Methods

### Experimental site and materials

A field study was conducted in 2016 at the agricultural experimental station of Yangtze University in Jingzhou, Hubei Province, central China (latitude 30° 21′N, longitude 112° 09′E, altitude 28 m). The site is in a typical subtropical monsoon climate zone with a mean annual precipitation of 1,095 mm. The mean annual sunshine duration is more than 1,718 h, and the mean annual temperature is 16.5 °C. The accumulated temperature of >10 °C is 5,094.9–5,294.3 °C. The soil pH was 7.4, total N 2.04 g kg^−1^, total P 0.48 g kg^−1^, available N 79.5 mg kg^−1^, available P 38.5 mg kg^−1^ and available potassium (K) 108.7 mg kg^−1^ in the plough layer (0–40 cm soil depth).

The experimental rice used was mid-maturation two-line hybrid rice (*Oryza sativa* L.) variety “Huifeng8”. The controlled-release N fertilizer used was CRF (N:P_2_O_5_:K_2_O = 28:5:9) (Kingenta Ecological Engineering Co., Ltd., Shandong, China) with 70 days of N released period. The two conventional N fertilizers used were urea (46% N) and common compound fertilizer (N:P_2_O_5_:K_2_O = 18:8:15). The P and K fertilizers used were superphosphate (12% P_2_O_5_) and potassium chloride (60% K_2_O), respectively.

### Experimental design

The experimental factors were comprised of the irrigation method and N fertilizer management. The irrigation treatments included conventional flooding irrigation (FI) and shallow-irrigation and deep-sluice (SIDS). The N treatments included local common N fertilization practice, 30% of fertilizer N (urea) mixed with the other 70% of controlled release N fertilizer (CRF), and optimized and reduced N fertilization, designated CN, U + CRF and ON, respectively. This experimental plan yielded six treatments. There were three replicates for each treatment.

At 10–14 days after the transplantation of rice, a standing water depth of 10–40 mm was maintained for all the treatments to facilitate recovery of seedlings and their ability to turn green. FI and SIDS were then managed differently. In FI, a standing water depth of 10–80 mm in the field was maintained until the terminal drainage at approximately 10 days before the harvest of rice. In SIDS, the field was allowed to be intermittently submerged and was not irrigated unless the standing water depth dropped to approximately 100 mm below the topsoil. The field was re-flooded to a standing water depth of 40–60 mm in each irrigation event. The cycles were repeated until the necessary drainage before harvest^14^. However, there was one week of exception to the drainage to maintain a standing water depth of 30–50 mm at the flowering stage. Wu *et al*.^28^ reported that the sluice water above soil surface of paddy field could be maintained up to 50, 100, and 150 mm at re-greening, tillering to jointing, and booting to maturity stages of rice, respectively. In the CN treatment, 70% N (common compound fertilizer) was applied at the basal, and 30% N (urea) was applied as topdressing at the tillering stage. In the U + CRF treatment, basal applications of 70% N used CRF, and the other 30% N used urea as N source were utilized. In the ON treatment, common compound fertilizer was applied at the basal (50% N), and the urea was top-dressed at tillering (35% N) and heading (15% N) stages, respectively. The corresponding dates were June 4, July 6, and August 1, 2016, respectively. The application of N fertilizer rate for CN, U + CRF and ON treatments was 180, 150 and 150 kg N ha^−1^, respectively. A total of 75 kg P_2_O_5_ ha^−1^ and 105 kg K_2_O ha^−1^ was utilized as a basal application for each N treatment. Details of the fertilization scheme, including fertilizer sources and the corresponding application rates used for each N treatment, are shown in Table 1.

**Table 1:** Fertilization scheme for rice under the different nitrogen fertilizer treatments.

Treatment	Basal application					Topdressing one	Topdressing two
	CRF (kg ha^−1^)	CCF (kg ha^−1^)	U (kg ha^−1^)	S (kg ha^−1^)	PC (kg ha^−1^)	U (kg ha^−1^)	U (kg ha^−1^)
CN	/	700	/	158	/	117	/
U + CRF	450	/	117	438	108	/	/
ON	/	417	/	347	70.8	114	49

Note: / represents that there was no fertilization.

S, superphosphate; U, urea; CRF, controlled-release nitrogen fertilizer; CCF, common compound fertilizer; PC, potassium chloride.

### Experimental management

The experimental field was planted with rice year round and served as a representative paddy field. Each plot was 30 m^2^ (6 m × 5 m), and the circumference of each plot was isolated by a PVC sheet that was 60 cm high (30 cm below the soil surface and 30 cm above the soil surface) to form a barrier. Each plot was separately irrigated and drained. The 4-week-old seedlings were artificially transplanted at 25 cm × 30 cm with 3 seedlings per plant on June 5, 2016. The plots were regularly hand-weeded to prevent weed damage during the rice grown season. Diseases and insects were intensively controlled by chemicals to avoid a loss of yield. The whole growth period was 136 d in all treatments. The rice was harvested on September 18, 2016. All of rice straw was returned to paddy field after the crop harvest. A water meter was installed at the discharging end of water inlet to measure amount of water applied, and a container was installed at the terminal of drainpipe to collect runoff.

## Measurements

### Precipitation

The amount of precipitation during the rice grown season was recorded by an automatic weather station (ICT, Australia) within the experimental base (approximately 100 m away from the plots).

### Runoff and leaching water

Before the experiment, a homemade iron leaching bucket (30 cm in diameter, 100 cm long) was installed vertically at a depth of 60 cm to collect the daily percolation water of each plot. The pipe orifice was 40 cm above the soil surface, and the upper part was covered to prevent rain, dust or insects from entering pipe. A water level indicator was used to measure water level at an interval of 2–3 days. The daily volume of percolation water in paddy fields was calculated according to the water level difference proposed by Yang *et al*.^23^. Ceramic suction cups were installed at 3, 9 and 12 m of the water inlet and vertically at a depth of 30 cm to measure leaching water after irrigation or precipitation (≥30 mm) during the growth stages of rice. After rainfall, the excessive water was drained on the basis of upper limit of sluice water at the different growth stages in SIDS. The runoff water was collected by an overflow bucket and sampled from the outlet of each plot. The amount of drainage was calculated from the difference between the water depth of the paddy field before and after drainage as described by Yang *et al*.^23^.

### N and P of water and soil

Soil samples were collected after the rice harvest using a stainless-steel auger. The S-shaped 5-point collection method was used for sampling. The samples were collected from 0–20 and 20–40 cm soil depths in each plot. Root debris and soil samples were sieved and stored as described by Si *et al*.^31^. In short, the plant root remnants and rocks were removed, and the samples were passed through a 0.85 mm sieve and stored at 4 °C to determine relevant indices.

The water quality parameters determined included the following: TP, TN, dissolved phosphorus (DP), ammonia-nitrogen (NH_4_^+^-N), nitrate-nitrogen (NO_3_^−^N) and particulate phosphorus (PP). Soil nutrient parameters that were monitored included TN, TP, NH_4_^+^-N, NO_3_^−^N and available P. All the analyses were conducted as recommended by the State Environmental Protection Administration of China (1997). Dissolved organic N (DON) was calculated by the differences between TN and sum of NH_4_^+^-N and NO_3_^−^N.

### Nutrient uptake

Five representative rice plants in each plot were cut at the re-greening, tillering, joint-booting, filling and maturity stages. The plants were divided into stems and leaves before heading stage, and stems, leaves and panicles after the heading stage. All the plant samples were oven-dried to a constant biomass at 70 °C and weighed. The samples were passed through a 0.15 mm sieve, and subsamples were collected for total N and total P determination as described by Ye *et al*.^2^. The total N was analyzed using the semi-micro Kjeldahl method, and the total P was analyzed using the vanadium molybdate yellow colorimetric method^32^. Tissue N or P concentrations were multiplied by the yield of dry matter to calculate total N or P uptake.

### Rice yield

Yield of grain was measured from a 5 m^2^ area in the center of each plot at maturity stage. The rice was harvested manually, and the grains were air dried.

### Data analysis

According to Zhu *et al*.^33^, the deep underground leaching was ignored owing to high water table (0.8–1.5 m below the surface) in the paddy fields at Jianghan Plain and the negligible plant interception (approximately 0.2% of precipitation amount). Precipitation use efficiency (*Y*) was calculated as follows:

1 $$Y=\frac{RT-{R}_{t}}{{R}_{T}}\times 100 \% $$

where *R*_*T*_ is the total amount of precipitation (mm) during a certain period of time, and *R*_*t*_ is the amount of runoff water (mm) during a certain period of time.

The amount of N or P loss was calculated as follows:

2 $$P=\mathop{\sum }\limits_{i=1}^{n}(Ci\times Vi)$$

where *C*_*i*_ is the concentration of N or P (mg L^−1^) in the runoff or leaching water at the *i* time, and *V*_*i*_ is the volume of runoff or leaching water at the *i* time (L).

An analysis of variance (ANOVA) was performed using the general linear model-univariate procedure from SPSS 12.0 software (USA). ANOVAs were conducted with irrigation method and fertilizer management as the main effects and included their interactions. The mean values were compared for any significant differences among different treatments using the Duncan’s multiple range tests at a significance level of *P* ≤ 0.05

## Results

In total, the leaching water was measured 20 times for SIDS and FI; the runoff water was measured four times for SIDS and seven times for FI during the rice growing season. The average of TN concentration from leaching water was 3.78 and 3.89 mg L^−1^ for FI and SIDS, respectively, which was comparable between the two irrigation regimes. However, the average of TN concentration from leaching water was 4.21 mg L^−1^ for the CN treatment, which was 18.7% and 20.3% significantly greater than that for the U + CRF and ON treatments, respectively. The average of TN concentration from runoff water and average of TP concentration from runoff and leaching water showed a highly similar amount of variation compared with the average of TN concentration from leaching water.

### Precipitation, runoff, irrigation and leaching water

**Figure 1:**
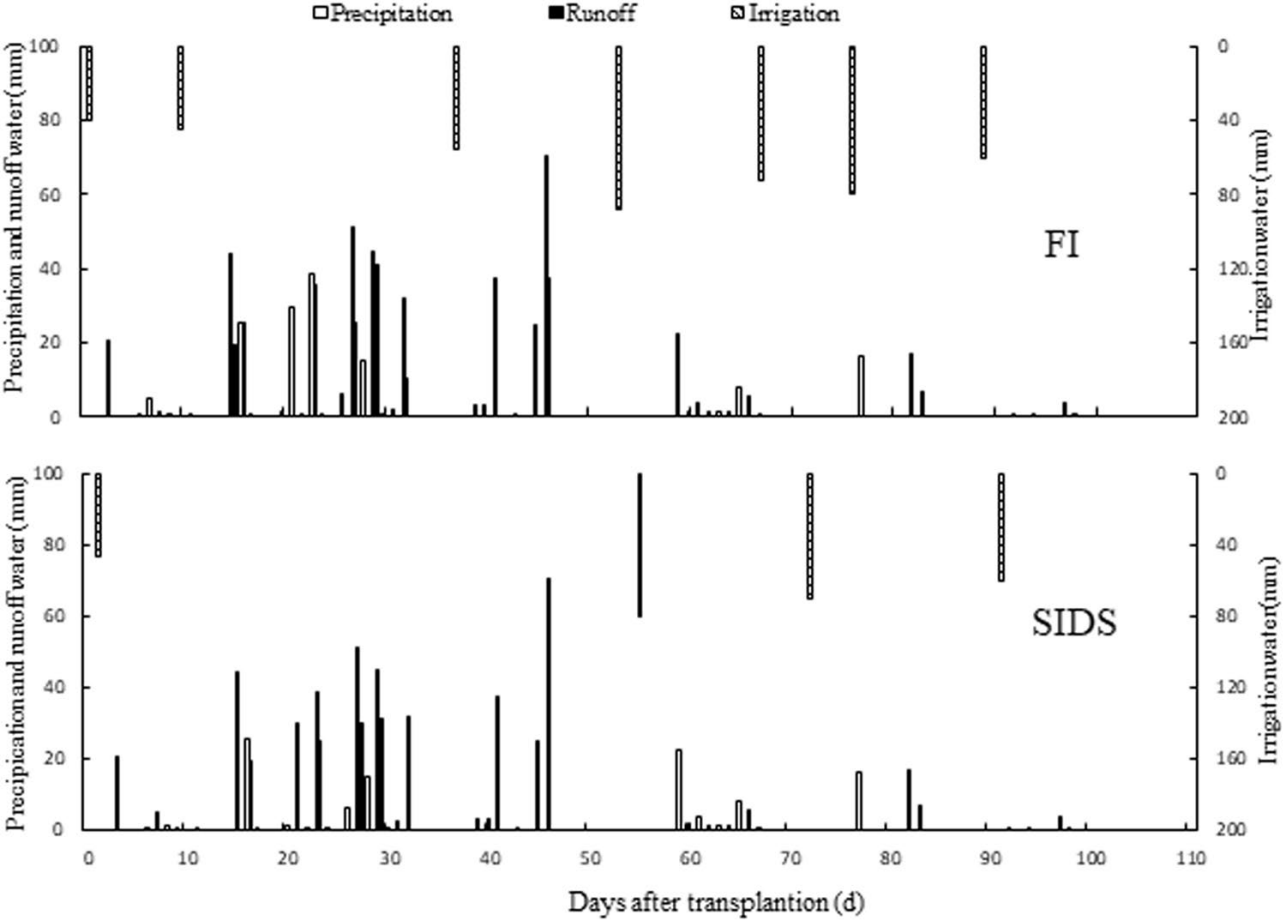
The volume of precipitation, irrigation and runoff water during rice grown season under continuously flooding (FI) irrigation and shallow-irrigation and deep-sluice (SIDS).

Within 110 days after the transplantation (DAT) of rice, the accumulated precipitation was 550.3 mm. The maximum daily precipitation of 70.3 mm was recorded on July 2, 2016 (Fig. 1). For the FI treatments, the field was irrigated seven times, and the amount of total irrigation water, total water consumption (sum of the accumulated precipitation and the total irrigation) and runoff water was 440.5, 990.8 and 194.8 mm, respectively. The runoff water of 157.3 mm was measured at tillering stage and accounted for 80.8% of total runoff water. The rest of total runoff water (37.5 mm) was measured at jointing-booting stage of rice. For the SIDS treatments, the field was irrigated four times, and the amount of total irrigation water, water consumption and runoff water was 257.0, 807.3 and 105.6 mm, respectively. The runoff water was only measurable at the tillering stage of rice. Compared with the FI treatment, the SIDS treatment significantly decreased the amount of irrigation water, total water consumption and runoff water by 41.7%, 18.5% and 45.8%, respectively, and significantly increased the precipitation use efficiency by 16.2% (Fig. 1). The amount of total leachate water from paddy field was 268 mm for SIDS and 343 mm for FI.

### Losses of N and P during the whole grown season

**Figure 2:**
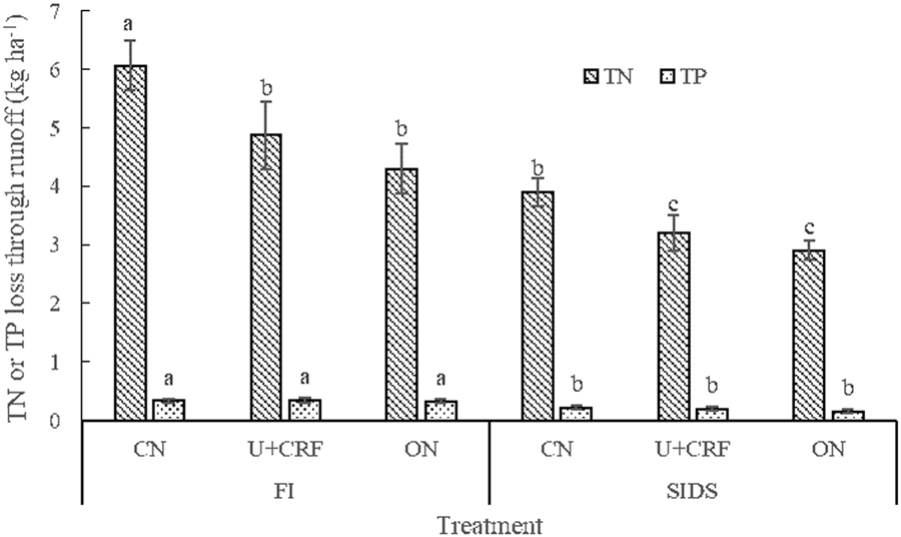
Total nitrogen (TN) and total phosphorus (TP) losses through runoff during the whole grown season of rice as affected by different water and nitrogen management strategies. Note: Values (mean ± standard error, n = 3) by different letters are significantly different at *P* < 0.05.

The FI treatments had the following runoff losses during the whole growth period of rice: NH_4_^+^-N 1.99–2.69 kg ha^−1^; NO_3_^−^N 0.77–1.16 kg ha^−1^; DON 2.69–4.87 kg ha^−1^; TN 4.30–6.07 kg ha^−1^; DP 0.14–0.16 kg ha^−1^; PP 0.17–0.19 kg ha^−1^; and TP 0.32–0.34 kg ha^−1^. Compared with the FI treatments, the runoff losses of NH_4_^+^-N, NO_3_^−^N, DON, TN, DP, PP and TP were 28.5–35.7%, 22.4–54.5%, 26.1–48.9%, 32.6–35.9%, 35.7–60.0%, 36.8–47.1% and 36.4–53.1% significantly smaller in the SIDS treatments, respectively. Both the U + CRF and ON treatments significantly decreased the loss of TN through runoff compared with that of CN treatment under the two irrigation treatments. FI coupled with CN resulted in the greatest runoff loss of TN, while SIDS coupled with U + CRF or CN resulted in the smallest one (Fig. 2). However, the runoff loss of TP was comparable among the different N treatments under FI and SIDS (Fig. 2). NH_4_^+^-N and DON were the major components of TN lost through runoff and accounted for 41.1–41.3% and 45.1–46.1% of the TN loss, respectively. PP was the major component of TP lost through runoff and accounted for 52.9–60.0% of the TP loss.

**Figure 3:**
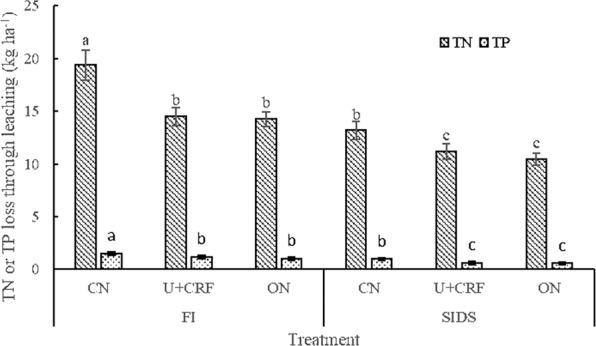
Total nitrogen (TN) and total phosphorus (TP) losses through leaching during the whole growth season of rice as affected by different water and nitrogen management strategies. Note: Values (mean ± standard error, n = 3) by different letters are significantly different at *P* < 0.05.

The FI treatments had the following amount of leaching losses during the whole growth period of rice: NH_4_^+^-N 8.98–13.83 kg ha^−1^; NO_3_^−^N 1.40–2.83 kg ha^−1^; DON 4.38–13.57 kg ha^−1^, TN 14.26–19.38 kg ha^−1^; DP 0.20–0.32 kg ha^−1^; PP 0.17–0.17 kg ha^−1^; and TP 0.37–0.49 kg ha^−1^. Compared with the FI treatments, the total amount of NH_4_^+^-N, NO_3_^−^N, DON, TN, DP, PP and TP lost through leaching was 23.5–28.1%, 12.9–37.5%, 16.8–28.8%, 22.8–32.0%, 5.0–36.4%, 23.5–29.4% and 16.2–33.3% significantly smaller in the SIDS treatments, respectively. Both the U + CRF and ON treatments significantly decreased leaching loss of TN compared with that of CN under the two irrigation treatments (Fig. 3). FI coupled with CN resulted in the greatest loss of TN by leaching, while SIDS coupled with U + CRF or CN resulted in the smallest loss of TN (Fig. 3). The loss of TP by leaching was very similar to that observed from the loss of TN by leaching (Fig. 3). NH_4_^+^-N was the major component of TN lost through leaching and accounted for 65.9–75.4% of the TN loss. DP was the major component of TP lost through leaching and accounted for 53.8–65.8% of the TP loss.

### Runoff losses of various forms of N and P

For FI, approximately 70% of the various N and P forms lost through runoff took place at tillering stage, and the other 30% took place at jointing-booting stage of rice. For SIDS, all of the various N and P forms lost through runoff took place at tillering stage (Table 2). Compared with FI, SIDS decreased the amount of NH_4_^+^-N, NO_3_^−^N, DP and PP losses. However, SIDS increased the amount of DON loss at U + CRF and ON (Table 2). Compared with the CN treatment, the U + CRF and ON treatments decreased amount of NH_4_^+^-N and NO_3_^−^N at tillering stage. The amount of DP and PP losses was comparable among the three N treatments under FI and SIDS (Table 2).

**Table 2:** Amount of various nitrogen (N) and phosphorus (P) form losses through runoff (kg ha^−1^) as affected by different water and nitrogen management strategies.

Growth stage	Index	FI			SIDS		
		CN	U + CRF	ON	CN	U + CRF	ON
Tillering	NH_4_^+^-N	2.27 a	1.53 b	1.54 b	1.73 b	1.43 c	1.28 c
	NO_3_^-^-N	1.07 a	0.90 b	0.73 b	0.90 b	0.66 b	0.35 c
	DON	1.42 a	0.77 c	0.73 c	1.26 a	1.11 b	1.27 b
	DP	0.11 a	0.12 a	0.12 a	0.09 b	0.08b	0.06 b
	PP	0.16a	0.15a	0.14a	0.12a	0.11a	0.09b
Jointing-booting	NH_4_^+^-N	0.43 a	0.47 a	0.45 a	/	/	/
	NO_3_^-^-N	0.09 a	0.06 b	0.04 b	/	/	/
	DON	0.79 a	1.14 a	1.02 a	/	/	/
	DP	0.03 a	0.03 a	0.03 a	/	/	/
	PP	0.03a	0.04a	0.03a			

Note: DON, dissolved organic nitrogen; DP, dissolved phosphorus; PP, particulate phosphorus. Means within a row followed by different letters are significantly different at *P* < 0.05.

### Leachate losses of various forms of N and P

The amount of various N and P forms lost through leaching during the re-greening to booting stages was approximately 75% of the whole rice growing stage. The other 25% was lost during heading to maturity. Compared with FI, SIDS decreased the amount of losses of NH_4_^+^-N, NO_3_^−^N and DON in each N treatment (Table 3). Compared with CN, the U + CRF and ON treatments decreased the amount of NH_4_^+^-N and NO_3_^−^N under FI and SIDS. However, the losses of DP and PP were comparable among different treatments (Table 3).

**Table 3:** Amount of various nitrogen (N) and phosphorus (P) form losses through leaching (kg ha^−1^) as affected by different water and nitrogen management strategies.

Growth stage	Index	FI			SIDS		
		CN	U + CRF	ON	CN	U + CRF	ON
Regreening-booting	NH_4_^+^-N	11.32 a	8.64 b	7.06 b	8.58 b	6.87 c	5.96 c
	NO_3_^−^N	1.92 a	1.26 a	0.85 a	1.48 a	1.00 ab	0.90 b
	DON	1.84b	1.38b	2.44a	0.95c	1.32b	1.72b
	DP	0.23 a	0.15 a	0.14 a	0.21 a	0.16 a	0.13 a
	PP	0.10 a	0.13 a	0.10 a	0.09 a	0.08 a	0.09 a
Heading-maturity	NH_4_^+^-N	2.51 a	1.88 b	1.92 b	1.36b	1.16c	0.91 c
	NO_3_^−^N	0.91 a	0.28b	0.55 ab	0.32 a	0.26 a	0.29 a
	DON	0.88c	1.05b	1.45a	0.52d	0.58d	0.61d
	DP	0.09 a	0.05 a	0.08 a	0.04 a	0.03 a	0.04 a
	PP	0.07 a	0.04 a	0.07 a	0.04 a	0.04 a	0.03 a

Note: DON, dissolved organic nitrogen; DP, dissolved phosphorus; PP, particulate phosphorus. Means within a row followed by different letters are significantly different at *P* < 0.05.

### N and P uptake by rice

As shown in Table 4, the N and P uptake by rice at re-greening and tillering stages was comparable between FI and SIDS treatments in each N  treatment with the exception of uptake of P in the ON treatment. Compared with FI, SIDS increased the uptake of N by rice at the jointing, filling and maturity stages in each N treatment. Similarly, SIDS treatments increased the P uptake by rice at jointing, filling and maturity stages in each N treatment. For the two irrigation regimes, compared with CN treatment, the U + CRF treatment increased the N and P uptake at all measured stages with the exception of uptake of P at the filling stage. The ON treatments increased uptake of N and P in general at the jointing, filling and maturity stages of rice. SIDS coupled with U + CRF achieved the greatest uptake of N and P by rice at maturity stage.

**Table 4:** Nitrogen (N) and phosphorus (P) uptake by rice (kg ha^-1^) as affected by different water and nitrogen management strategies.

Irrigation method	Fertilizer management	Re-greening		Tillering		Jointing		Filling		Maturity	
		NU	PU	NU	PU	NU	PU	NU	PU	NU	PU
FI	CN	1.12a	0.13ab	41.53a	2.91b	61.19b	12.31b	124.51c	30.40a	153.64c	48.86b
	U + CRF	1.40a	0.23a	49.40a	5.49a	73.14a	13.56b	157.55a	25.13b	171.87b	55.96b
	ON	1.05a	0.13b	26.21b	3.38b	70.59a	13.13b	138.82b	20.68a	154.79c	53.82b
SIDS	CN	1.13ab	0.16ab	41.66a	3.39b	71.13a	14.85b	137.51b	34.82a	157.37c	52.27b
	U + CRF	1.21a	0.19a	56.99a	6.39a	82.95a	16.78a	158.11a	30.38a	179.02a	63.29a
	ON	0.92ab	0.10b	30.23b	5.37a	76.06a	15.90a	139.00b	30.93a	167.72b	57.68b

Note: Means within a column followed by different letters are significantly different at *P* < 0.05.

NU, nitrogen uptake; PU, phosphorus uptake.

### Residual soil N and P after the rice harvest

**Figure 4:**
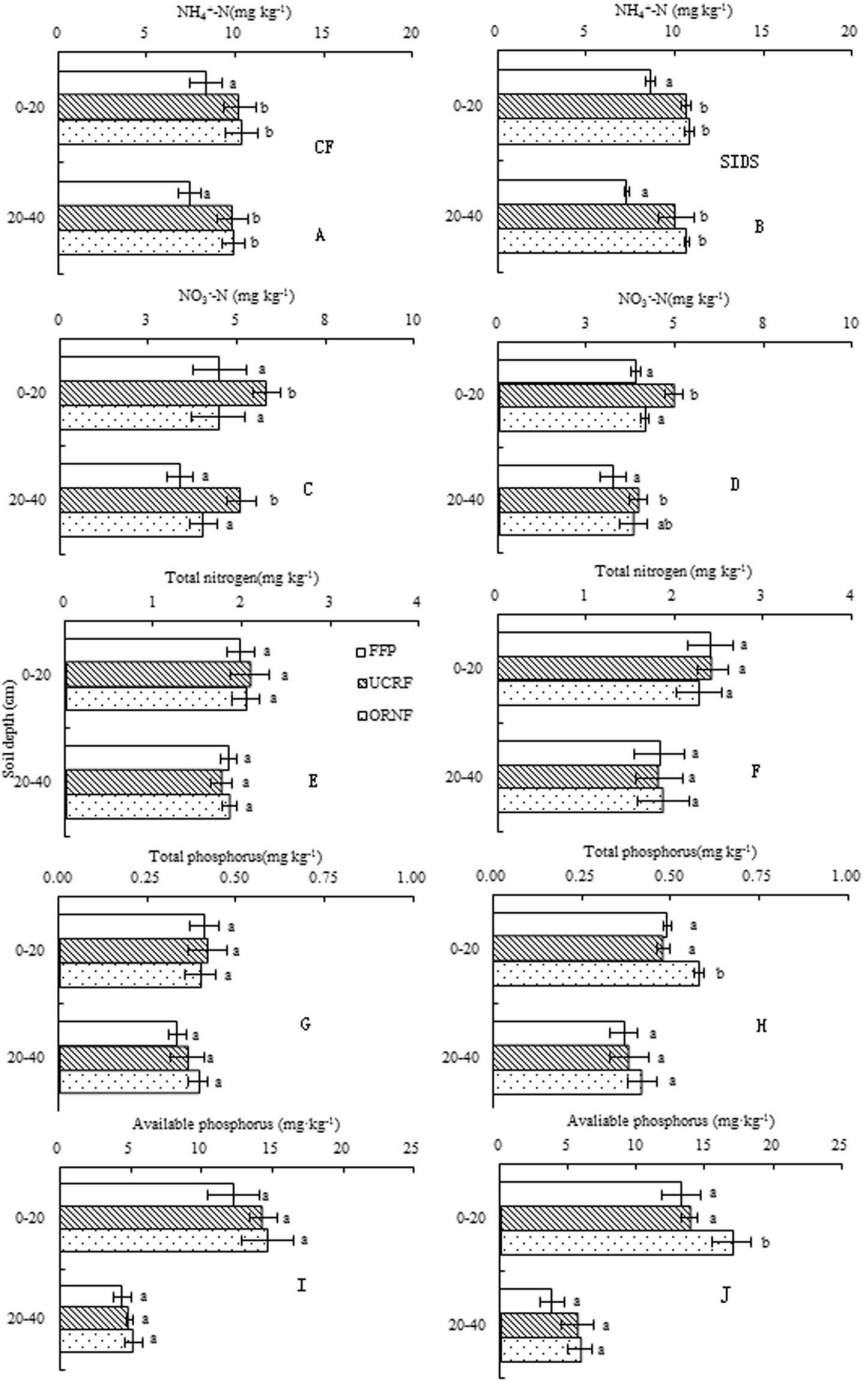
Soil NH_4_^+^-N, NO_3_^−^N, total nitrogen, available phosphorus and total phosphorus content in the 0–40 cm soil depths after rice harvest as affected by different water and nitrogen management strategies. Note: Values (mean ± standard error, n = 3) within the same soil depth and item followed by different letters are significantly different at *P* < 0.05.

As shown in Fig. 4, the amounts of soil NH_4_^+^-N, NO_3_^−^N, TN, available P and TP content in the 0–40 cm soil depths were comparable between the FI and SIDS treatments for the three N treatments. For the two irrigation regimes, the content of NH_4_^+^-N in the 0–40 cm soil depths was significantly greater in the U + CRF and ON treatments than that in the CN (Fig. 4A,B). The content of NO_3_^−^N in the 0–40 cm soil depths was greater in U + CRF than in the other N fertilizer treatments (Fig. 4C,D). The contents of TN and TP in the 0–40 cm soil depths were comparable among three N fertilizer treatments with the exception of TP content in the 0–20 cm soil depth under SIDS (Fig. 4E–H). The U + CRF and ON treatments increased soil available P content compared to FFP under FI and SIDS (Fig. 4I,J).

### Yield of rice

**Figure 5:**
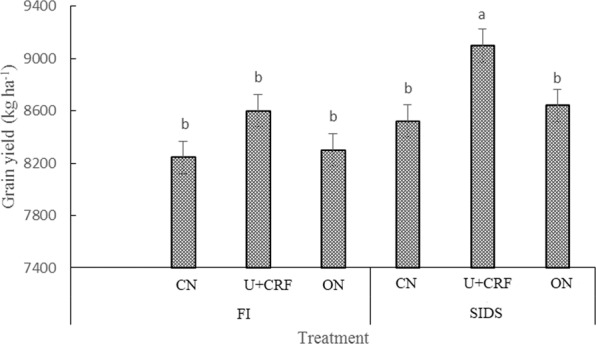
Grain yield of rice as affected by different water and nitrogen management strategies. Note: Values (mean ± standard error, n = 3) by different letters are significantly different at *P* < 0.05.

As shown in Fig. 5, the SIDS treatments increased yield of rice grains compared with the FI treatments, although a significant difference was only observed from the U + CRF treatment. The U + CRF resulted in a greater yield of grain compared with the other N treatments under SIDS. SIDS coupled with U + CRF resulted in the greatest yield of grain among the different treatments.

## Discussion

### N and P losses from paddy fields

Alternate wetting and drying irrigation could slightly increase the concentration of N compared with conventional flooding irrigation^21^. Consistently, SIDS resulted in an average of 2.9% higher TN concentration from leachate water compared with FI. This could be related to the smaller water depth of field and irrigation amount under water-saving irrigation, which led to an increase in substrate concentration^34^.

Earlier studies have shown that the amount of N and P lost through runoff and leaching are closely related to the water management measures^23,35^. Greater amounts of irrigation lead to more substantial nutrient losses through runoff and leaching from a paddy field^23^. In this study, the SIDS treatments significantly reduced the irrigation frequency by 42.3% and amount of irrigation by 41.7%, leading to the lower runoff and leaching water (reduced by 45.8% and 21.9%, respectively). This resulted in the significantly reduced amount of TN and TP lost through runoff and leaching under SIDS (Figs. 2 and 3), which is consistent with the findings of Liang *et al*.^36^ derived from alternate wetting and drying irrigation. This confirms the substantial water-saving and environmental protection effects of SIDS^13,14^. The possible reasons include the fact that field water depth is an important factor that contributes to occurrence of surface runoff and deep leaching from paddy fields^2,20,21^. A high field water depth results in more surface runoff when faced with concentrated or heavy precipitation^2^. SIDS significantly decreased the field water depth compared with FI (data not shown) and thereby decreased the occurrence and amount of surface runoff (Fig. 1), resulting in the increased use of precipitation and reduction in losses of N and P. Moreover, the low water level and large air exchange capacity at water-soil interface under SIDS helped to increase the P fixation by soil. Fe^2+^ is oxidized to Fe^3+^, and then Fe^3+^ easily combines with PO_4_^3−^ to form insoluble Fe(PO_4_)^37^. In addition, Liang *et al*.^36^ suggested that alternate wetting and drying irrigation reduced the irrigation water, runoff water and TN loss through runoff by 13.4–27.5%, 30.2–36.7% and 23.3–30.4%, respectively, in comparison with FI, which were lower than the corresponding values obtained from this study. This could be related to the ability of SIDS to more effectively use precipitation compared with alternate wetting and drying irrigation^13^, thereby enhancing the WUE and reducing losses of nutrients. In addition, the reduced frequency of irrigation results in a more economical use of the labor force^38^.

An earlier study has shown that there was a substantial difference in the loss of TN through runoff during rice growing season, which ranged from 0.5 to 54.3 kg ha^−1,^^39^. The variation was associated with differences in precipitation, soil types, crop growth conditions and management of irrigation water and mineral fertilizers^23,36^. In this study, the loss of TN through runoff during the rice grown season was 2.90–6.07 kg ha^−1^ among different treatments, which are relatively low levels^39^. Nevertheless, our measured losses are within the range of those in other studies that quantified losses of N from paddy fields^21,23^. This was primarily attributed to the low total runoff volume (105.6–194.8 mm). Moreover, the time of runoff events was long behind N fertilization (>8 d), resulting in the low N concentration in the surface water of paddy fields. It has been shown that the amount of N lost through runoff can be significantly reduced when the runoff occurred one week after the N fertilization^34^.

In this study, the U + CRF and ON treatments reduced loss of TN through runoff by 19.7–29.2% and 25.4–51.7%, respectively, and loss of TN through leaching by 15.1–25.2% and 20.9–26.4%, respectively, in comparison with the CN treatment (Fig. 2). These results are consistent with the findings of Yang *et al*.^23^ and Ji *et al*.^40^ Because controlled-release N fertilizer has the characteristics of “peak cutting and valley filling,” namely its N release amount is not too much at the early growth stages and not too small at the later growth stages of rice^30,41^, this effect was conducive to improving the metabolism of N in rice^40^, which corresponded to the increased N uptake (Table 4) and decreased TN concentration from leaching and runoff water. Consequently, this reduced the loss of N. In terms of the ON, numerous studies have shown that the optimal application of fertilizer can reduce concentration of nutrients from source^42,43^, which is consistent with our results. Moreover, an earlier study has shown that TN leaching increases in a significantly linear fashion in parallel with the increase in rate of application of N in a typical open field for vegetable planting^26^. A 40% reduction in traditional N rate of synthetic fertilizer could reduce amount of NO_3_^−^N leaching by 39.6%^44^. Thus, these results strongly suggest that appropriately reducing fertilizer inputs can be an efficient approach to reduce loss of N from paddy fields. In addition, both the U + CRF and ON treatments could partially avoid adverse effects of plum-rain season owing to relatively low concentration of N in paddy water, resulting in a reduction in loss of N. Most dramatically, SIDS coupled with the U + CRF or ON treatments achieved the smallest N and P losses through runoff and leaching, suggesting that SIDS coupled with U + CRF or ON can help reduce the N and P losses through runoff and leaching from paddy fields.

The U + CRF and ON treatments reduced loss of TP through leaching by 18.4–24.5% and 20.4–31.6%, respectively, compared with that of the CN treatment (Fig. 3). This was attributed to the lower concentration of P in the leachate water. It has been shown that the concentration of P in leachate water increased with increase in rate of N fertilization^21^. Since the N fertilization could occupy the soil colloid or iron and aluminum oxide surface adsorption and decrease the ability of soil to absorb P, this type of fertilization could result in the enhancement of dissolution of P and its release into water^45^. In the U + CRF treatment, the controlled release N fertilizer enabled the process of release of N to become slower and longer compared to that of CN^46^. In the ON treatment, the rate of N fertilizer input was reduced by approximately 17%. As a result, both the U + CRF and ON treatments resulted in a lower concentration of N in leachate water. In addition, Peng *et al*.^30^ found that controlled-release N fertilizer reduced the loss of TN through leaching by 53.6%, which was far greater than the corresponding data in this study, which was 18.4–24.5%. This was attributed to the N rate of controlled release N fertilizer treatment being far smaller than that of standard fertilization by the farmer (180 vs 403 kg N ha^−1^) in Peng’s study^30^, but the same N rate was used for the U + CRF and CN treatments (180 vs 180 kg N ha^−1^).

In this study, NH_4_^+^-N was the major component of loss of TN through leaching (Table 3). This is consistent with the findings of Peng *et al*.^30^ and Ji *et al*.^47^. Owing to long-term flooding and the high groundwater table in paddy fields at Jianghan Plain (less than 150 cm below the surface), the paddy soil is maintained in a reduced state^23^. The anaerobic environment inhibits the activity of autotrophic nitrifying bacteria, resulting in limited soil nitrification. Moreover, ammonization, denitrification, and biological nitrogen fixation are the three primary forms of N transformation in flooded soil, with the result that most inorganic N exists in the form of NH_4_^+^-N^21,48^. Moreover, PP was the major component of TP loss through runoff (Table 2). This is consistent with the findings of Liang *et al*.^49^ and Ye *et al*.^20^. Because precipitation or irrigation would impact the soil surface, which causes a substantial amount of PP in the soil to move to paddy water and consequently be lost through runoff. However, DP was the major component of loss of TP through leaching (Table 3). This was attributed to the fertility of paddy soil in Jianghan Plain and the generally high contents of clay and organic matter in soil^27^, leading to the strong adsorption and filtration function of PP in percolating water.

### N and P uptake by rice and rice yield

The optimization of water management can realize the purpose of promoting effect of fertilizer with water, which is of substantial importance on the efficiency of improvement of water and fertilizer use to achieve a stable and high yield of rice^50^. In this study, the SIDS treatments resulted in a higher uptake of N and P at maturity and yield of rice grain compared with those of FI treatments (Table 4, Fig. 5). This can be explained as follows: SIDS enhanced the air exchange between soil and the atmosphere owing to the alternate wetting and drying cycles^11,51^. Therefore, the root system was surrounded by relatively sufficient oxygen to accelerate mineralization of soil organic matter and inhibit soil N immobilization, resulting in the increased soil available nutrients for rice growth^52,53^. It has been shown that water-saving irrigation resulted in higher activities of glutamine synthetase, glutamate synthase, and glutamate dehydrogenase (the main enzymes involved in plant N metabolism) compared to conventional flooded irrigation^7^. Therefore, the root growth, N metabolism, and photosynthetic rate in leaves of rice were improved under SIDS, leading to a high yield of grain^54,55^.

Optimal fertilization measures help to improve the uptake of nutrients, yield of grain, conserve soil nutrients and reduce excessive nutrient loss to prevent water eutrophication^19^. In this study, the U + CRF and ON treatments resulted in comparable TN and TP contents in the 0–40 cm soil depths after rice harvest (Fig. 4) and resulted in a better or comparable uptake of N and P at the latter growth stages (Table 4). These results indicate that the U + CRF and ON treatments improved uptake of N and P by rice and did not lead to excessive accumulation of TN and TP in soil. Similar results were also reported from other studies^21,35^. The increased uptake of N and P can be explained as follows: in the U + CRF treatment, the use of controlled-release N fertilizer prolonged the amount of soil available N at latter growth stages of rice, which is conductive to promote effective panicles per m^2^ and spikelets per panicle, and thereby enhance the uptake of N^2^. In the ON treatment, postponing and decreasing the application of N fertilizer reduces the transfer rate of N in leaves and delays leaf senescence, resulting in a high accumulation of N in rice^56^. In addition, approximately reducing the rate of fertilizer usage could enhance uptake of N and P by rice^57^. The relatively lower TN and TP losses through leaching under ON (Fig. 3) supported this conclusion. Thus, it is no surprise that the U + CRF treatment significantly increased yield of grain, and the ON treatment maintained yield of grain compared with that of CN treatment (Fig. 5). Higher grain yield obtained under controlled release N fertilizer than under urea with an equivalent rate of N had been reported previously^2,58^. Most dramatically, compared to FI coupled with CN, SIDS coupled with U + CRF or ON resulted in a better or comparable uptake of N and P (Table 4) and yield of rice grains (Fig. 5). These results suggest that SIDS coupled with U + CRF or ON was effective at improving or maintaining the N and P uptake and grain yield of rice.

This study strongly illustrates that water-saving irrigation can reduce the losses of N and P from runoff and leaching from paddy fields owing to decreasing amounts of runoff and water. Controlled release N fertilizer and optimized and reduced N fertilization reduce the losses of N and P by lowering concentrations of N and P in the runoff and leaching water. Thus, the combination of SIDS and U + CRF or ON obviously reduced N and P losses during rice grown season. In the future, the mechanism of reducing N and P losses from SIDS coupled with new N management should be considered from perspective of soil N and P cycles.

## Conclusions

This study demonstrated that SIDS enabled the paddy field to receive fewer irrigation frequencies (a decrease of 42.3%) and less irrigation water (savings of 41.7%) while using more water from precipitation (an increase of 16.2%), resulting in the reduced total amount of surface runoff and leaching water by 45.8% and 21.9%, respectively. Consequently, SIDS significantly reduced the losses of TN and TP through runoff and leaching. The U + CRF and ON treatments generated a lower loss of TN through runoff and leaching and loss of TP through leaching compared with that of CN treatment. The combined SIDS and U + CRF or ON reduced the N and P loss through runoff and leaching and enhanced or maintained N and P uptake at the later growth stages of rice, subsequently improving the yield of rice grains.
